# Do age, gender, and subjective health-related factors influence health-related life satisfaction in people with disabilities who are physically active?: a secondary analysis

**DOI:** 10.4069/whn.2024.03.18

**Published:** 2024-03-29

**Authors:** Hyunseok Cho, Sukhee Ahn

**Affiliations:** College of Nursing, Chungnam National University, Daejeon, Korea

**Keywords:** Age groups, Disabled persons, Exercise, Gender equity, Health

## Abstract

**Purpose:**

This study explored the factors influencing the health-related life satisfaction of people with disabilities who engaged in physical activity, by age and gender.

**Methods:**

A secondary analysis was conducted of the 2020 Third Disability and Life Dynamics Panel (2021). The participants were 2,796 people who performed regular physical activity at least once a week. The variables selected were disability-related factors (degree of disability, multiple disabilities, and type of disability), sociodemographic factors (age, gender, living alone, and mean monthly family income), and health-related factors (amount of physical activity, self-esteem, depression, chronic disease, subjective health, and health-related life satisfaction). Descriptive statistics, the chi-square test, the t-test, two-way analysis of variance, and multiple regression analysis were conducted.

**Results:**

In total, 58.0% of participants were male, and 42.0% were female. For age groups, 14.4% were children/adolescents (0–19 years), 42.6% were adults (20–59 years), and 43.0% were seniors (≥60 years). The mean score for health-related life satisfaction was 5.0±2.15 out of 10. Adults and seniors whose level of physical activity met or exceeded recommendations had higher subjective health. Moreover, men had better subjective health than women in seniors. Health-related life satisfaction was higher among those who had higher self-esteem, were not depressed, did not have chronic diseases, and had better subjective health.

**Conclusion:**

Gender significantly influenced health-related life satisfaction in children/adolescents and seniors. Disability-related factors were significant in adults, and health-related factors were significant in all age groups. Therefore, these factors should be considered when designing interventions to promote subjective health and health-related life satisfaction of people with disabilities.

## Introduction

Subjective health has recently become a significant factor in health-related research [1]. Over 50% of individuals with disabilities perceive their health negatively [2], in contrast to 18.4% of those without disabilities [3]. The subjective health gap between disabled and nondisabled individuals has narrowed slightly, from 35.46% in 2011 to 31.02% in 2018. Notably, this gap is smaller among those over 65 years of age than in the 18 to 64-year age group [4]. This may be due to the difficulty in differentiating between the concepts of “aging with a disability” and “disability with aging” in the older disabled population [5]. Women reported a more negative subjective health status (59%) than men (43.3%) [6]. Thus, both gender and age are significant factors in how people with disabilities perceive their health [7]. Additionally, individuals with external functional disabilities tend to view their health as worse than those with internal functional disabilities [2].

Engagement in physical activities has been linked to improved subjective health and life satisfaction among people with physical disabilities [8,9]. Individuals with disabilities participate in medium-intensity physical activities (20.2%) more frequently than those without disabilities (16.6%) [10]. However, the intensity and volume of physical activity vary according to the type and severity of the disability. People with visual impairments have the highest rate (30.8%) of regular daily exercise, while those with cerebral palsy have the lowest (12.3%). Similarly, those with mild disabilities (27.9%) exercise more regularly than those with severe disabilities (19.3%) [11]. Physical activity levels among people with disabilities also differ by gender and age, with males reporting more exercise in the past year than females [11,12]. Those in their 50s (50.3%) are the most active, while those in their 30s (45.9%) are the least [11]. During the coronavirus disease 2019 (COVID-19) pandemic, the annual exercise rate among people with disabilities fell from 77.0% in 2019 to 49.4% in 2020, and further to 39.6% in 2021. This reduction in physical activity among the disabled population is concerning, as it suggests a potential decline in health due to decreased physical function [11,13,14].

High self-esteem is a pivotal contributor to success in various life domains; therefore, boosting self-esteem is crucial for individuals and society [15]. However, studies have shown that individuals with disabilities tend to have lower self-esteem than those without disabilities, a disparity that persisted both before and after the emergence of COVID-19. It is important to note that self-esteem levels have further decreased after the onset of COVID-19, particularly among individuals with physical disabilities and those younger than 65 years [16]. On a positive note, people with physical disabilities who participate actively in sports have reported higher self-esteem and greater life satisfaction [17].

Depression is associated with lower life satisfaction among individuals with disabilities [18]. Specifically, those with disabilities who experienced depression as a result of COVID-19 were found to have a 1.6-fold higher risk of reduced life satisfaction than individuals without disabilities [19].

Chronic diseases are recognized as exacerbating factors that negatively impact the health of individuals with disabilities, more so than those without disabilities [20]. Furthermore, sociodemographic factors, including cohabitation status and economic level, have been linked to the health and life satisfaction of people with disabilities. Life satisfaction is greater among young people with disabilities when they are part of larger households [21]. Conversely, older individuals with disabilities living alone tend to have lower overall health compared to those in multi-person households [22]. Additionally, a higher income level correlates with increased life satisfaction in middle-aged and older people with disabilities [23].

In summary, prior research has established that the health and life satisfaction of individuals with disabilities is influenced by factors related to disability [2], sociodemographics [7,21-23], and health [8,9,17-20]. However, the majority of these studies have examined the impact of each factor in isolation and have been confined to particular age groups and disability types.

This study investigated the impact of disability-related, sociodemographic, and health-related factors on health-related life satisfaction in people with disabilities, according to age and gender. The specific aims were as follows:

1) To identify disability-related and sociodemographic characteristics by age and gender

2) To compare health-related life satisfaction and health-related factors by age and gender

3) To examine differences in subjective health and health-related satisfaction between genders and amount of physical activity among different age groups

4) To analyze factors affecting health-related life satisfaction among different age groups

## Methods

**Ethics statement:** This study did not require Institutional Review Board approval or informed consent because it was a secondary analysis of panel survey data from the Korea Disabled People’s Development Institute and the data were anonymized.

### Research design

This secondary data analysis was conducted using a descriptive correlational study design. The original dataset was the 2020 third Disability and Life Dynamics Panel (2021), conducted by the Korea Disabled People’s Development Institute.

### Study participants

According to the Disability and Life Dynamics Panel user guide (1st to 3rd) [24], the survey’s target population consists of people identified as disabled under Article 2 of the Disabled Welfare Act and their household members, from 2015 to 2017. From the 5,259 respondents with disabilities, this study focused on 2,796 people who reported engaging in regular physical activity at least once a week. There were no missing data, and all 2,796 respondents’ data were analyzed.

### Measurements

#### Health-related life satisfaction

The question of health-related life satisfaction was scored from “very dissatisfied (1 point)” to “very satisfied (10 points)”.

#### Health-related factors

##### (1) Amount of physical activity

The metabolic equivalent of task (MET) quantifies physical activity by setting the baseline oxygen consumption at rest as 1 MET and expressing the oxygen consumption of various physical activities as multiples of this baseline [25]. This metric was derived by assessing the types of exercises participants regularly engaged in, the average number of days per week they exercised, the frequency of their daily exercise sessions, and the duration of each exercise session. The type of exercise performed indicated the intensity level of the physical activity. Utilizing the Korean version of the Global Physical Activity Questionnaire (GPAQ) [26] and the Physical Activity Classification Table for Koreans [27], we classified the exercise type responses into two categories: moderate-intensity physical activity (4.0 METs) and high-intensity physical activity (8.0 METs). Following the World Health Organization (WHO) GPAQ analysis guidelines [28], we calculated metabolic equivalent work minutes per week (MET-min/week), which is a continuous variable. The recommended physical activity levels are 1,680 to 3,360 MET-min/week for children and adolescents aged 5 to 17 years, and 600 to 1,200 MET-min/week for adults and older adults [29,30]. Therefore, we operationalized the amount of physical activity as “meeting or exceeding recommendations” and “less than recommended” based on each age group’s criteria.

##### (2) Self-esteem

Self-esteem was measured using the Rosenberg Self-Esteem Scale [31], which was translated into Korean by Jeon [32]. This scale consists of 10 items, each rated on a 4-point Likert scale ranging from 1 (strongly disagree) to 4 (strongly agree). Higher scores, with a possible range of 10 to 40, indicate greater self-esteem. The Cronbach’s alpha for the scale was reported to range from .75 to .87 by Lee et al. [33], and it was .77 in the current study.

##### (3) Depression

Depression was measured using the 11-item Center for Epidemiologic Studies Depression Scale [34], which is rated on a 4-point Likert scale. For example, a score of 0 indicates that during the past week the respondent rarely or never experienced symptoms (less than 1 day), while a score of 3 indicates symptoms were experienced all the time (5–7 days). The total score can range from 0 to 33, with higher scores indicating more severe depression. The Cronbach’s alpha for this scale was reported to be .906 [35], and in this study, it was .882. Following the user guide of the Disability and Life Dynamics Panel of the Korea Disabled People’s Development Institute [36], the total depression score can be converted to a 0 to 60 scale. Generally, a score of 16 or higher is considered indicative of depression [37]. Therefore, in this study, participants were categorized as depressed (≥16) or not depressed (<16) based on this threshold.

##### (4) Chronic diseases

This study classified participants as having a chronic disease if they reported any of the 20 categories of chronic diseases, which include conditions such as cancer, arthritis, and gastritis. Conversely, participants were categorized as having no chronic diseases if they did not report any conditions from the list.

##### (5) Subjective health

Subjective health status was assessed using a single item that reflected overall health over the past 6 months. Participants rated their health on a scale ranging from “very bad (1 point)” to “very good (4 points)”.

#### Characteristics related to disabilities

Disabilities were categorized by type, degree (mild or severe), and the presence of multiple disabilities. Of the 15 types of disorders, six—namely hearing impairment, language impairment, cerebral palsy, physical disability, visual impairment, and facial disorders—were classified as external physical functional disabilities [38]. The remaining nine, which included intellectual disability, autistic disorder, mental disorder, and disorders of internal organs such as the heart, liver, and kidney, were categorized as internal organic functional or mental disabilities.

#### Sociodemographic factors

The sociodemographic factors analyzed in this study were age, gender, living alone, and the monthly family income. Age was divided into three categories: children/adolescents (0–19 years), adults (20–59 years), and seniors (60 years and older). Gender was determined by self-identification and categorized as either male or female. Based on the response to the household size question, those who reported living in a one-person household were categorized as living alone’ while all other responses were categorized as living with someone. This study utilized mean monthly household income data, which was obtained from the head of the household’s information by matching the panel data with specific codes. As outlined in the Disability and Life Dynamics Panel user guide, the reference year for calculating the mean monthly income of households is the year prior to the survey. This income data encompassed the total earnings from various sources, including labor, business and side jobs, personal property, finances, pensions, private transfers, public transfers, and non-recurring income. In instances where respondents did not provide a mean value for their monthly income in a particular subsection, the data was computed using the median of the income categories that were provided.

### Data analysis

The data were analyzed using the IBM SPSS ver. 26.0 (IBM Corp., Armonk, NY, USA), with the significance level set at .05. The chi-square test or t-test was used to examine differences in disability-related sociodemographic and health-related factors by age and gender. Two-way analysis of variance was employed to analyze differences in subjective health and health-related life satisfaction between the levels of physical activity and gender across various age groups. Finally, multiple regression analysis was conducted to identify the factors influencing health-related life satisfaction.

## Results

### Disability-related and sociodemographic characteristics by age and gender

Of the 2,796 participants, 58.0% were male and 42.0% were female. The age distribution included 404 children/adolescents (14.4%), 1,190 adults (42.6%), and 1,202 seniors (43.0%). A higher proportion of cases involved external physical functional disability (64.6%) compared to internal organic functional or mental disability (35.4%). Among children/adolescents, females exhibited a higher incidence of mild disability than their male counterparts (65.7% vs. 54.5%; χ²=4.69, *p*=.030). Approximately 7% of all respondents had multiple disabilities, with a higher prevalence among male adults than females (6.8% vs. 3.8%; χ²=5.40, *p*=.020). Regarding living alone, a larger percentage of male adults lived alone compared to females (25.2% vs. 16.5%; χ²=13.22, *p*<.001). The mean monthly family income was also higher for males than for females among children/adolescents (t=2.30, *p*=.022) (Table 1).

### Health-related life satisfaction and health-related factors by age and gender

The level of health-related life satisfaction was at the midpoint, with a mean of 5.00 (standard deviation [SD], ±2.15). The score was higher among female children/adolescents (t=2.31, *p*=.021) and male seniors (t=3.90, *p*<.001). Participants’ physical activity levels varied widely, ranging from 40 to 47,040 METs-min/week, with a median of 720.0 METs-min/week. A slightly higher proportion of participants engaged in physical activity to an extent that met or exceeded recommendations (52.6%) than those with less than recommended levels (47.4%). Gender differences in physical activity were observed in adults and seniors, with a higher proportion of females than males falling into the “less than recommended” category (χ²=3.93, *p*=.047 in adults; χ²=15.46, *p*<.001 in seniors). Approximately 43% of the participants were experiencing depression, with female adults reporting higher levels of depression than males (χ²=4.36, *p*=.037). Over half of the participants (57.2%) had a chronic disease, with female adults and seniors showing a higher prevalence of chronic diseases than their male counterparts (χ²=7.17, *p*=.007; χ²=7.22, *p*=.007, respectively). The mean score for subjective health was 2.60 (SD, ±0.57), which was above the midpoint. Male seniors reported higher subjective health scores than females (t=2.98, *p*=.003) (Table 2).

### Differences in subjective health and health-related life satisfaction according to the amount of physical activity and gender across age groups

Both adults and older individuals who engaged in physical activity that met or exceeded recommendations reported significantly better subjective health (F=9.94, *p*=.002 for adults; F=5.25, *p*=.022 for older individuals). Male older individuals reported higher subjective health than their female counterparts (F=8.89, *p*=.003). Similarly, adults and older individuals who performed physical activity that met or exceeded recommendations experienced significantly higher health-related life satisfaction (F=5.58, *p*=.018 for adults; F=6.10, *p*=.014 for older individuals). Male seniors reported higher health-related life satisfaction than females (F=15.24, *p*<.001) (Table 3).

### Factors affecting health-related life satisfaction by age

Regression model 1 included sociodemographic and disability-related factors and model 2 added health-related factors to explain health-related life satisfaction.

For children/adolescents, model 1 was insignificant (F=1.29, *p*=2.57), but model 2 was statistically significant (F=16.82, *p*<.001) with an explanatory power of 30.2%. Health-related life satisfaction among children and adolescents was greater in females, those with higher self-esteem, those not experiencing depression, and those with better subjective health.

For adults, model 1 identified multiple significant factors: having a mild disability, not having multiple disabilities, living with someone, and higher family income (F=11.15, *p*<.001). Model 2 was significant (F=55.41, *p*<.001), with an explanatory power of 33.5%. Thus, adults with a mild disability, no multiple disabilities, higher self-esteem, no depression, no chronic diseases, and higher subjective health had higher health-related life satisfaction.

For seniors, model 1 showed the following statistically significant factors: male gender, having a mild disability, not having multiple disabilities, and higher family income (F=13.37, *p*<.001). Model 2 was also significant (F=58.73, *p*<.001), with an explanatory power of 34.6%. Thus, seniors with higher self-esteem, no depression, no chronic diseases, and better subjective health had higher health-related life satisfaction (Table 4).

## Discussion

This study analyzed the factors affecting health-related life satisfaction among people with disabilities by age and gender. The findings revealed that both disability-related and health-related factors significantly influence health-related life satisfaction.

This study found that female children/adolescents exhibited more severe disabilities than males. In contrast, data from “*Haengbok-E-eum*,” South Korea’s social security information system, suggest that males experienced more severe disabilities than females [39]. Furthermore, a prior study indicated that men between the ages of 15 and 64 years had higher disability severity than women [40]. The divergence between the results of this study and previous findings could be attributed to the fact that the current study concentrated on individuals with disabilities who engaged in physical activity.

Male adults and seniors engaged in more physical activity than their female counterparts. This result is consistent with two previous studies [6,41].

The study found no significant differences in self-esteem related to age or gender. This finding is in contrast to a prior study [41], which reported that females with disabilities exhibited lower self-esteem than their male counterparts, and that older individuals with disabilities had lower self-esteem than younger ones. The discrepancy between these findings may stem from the fact that the previous study considered people with disabilities as a whole, whereas the current study specifically targeted those who are physically active. In line with this, existing literature has demonstrated that individuals with disabilities who are physically active tend to report higher self-esteem than those who are not [42]. Furthermore, engaging in physical activity has been shown to improve the self-esteem of people with disabilities [43,44].

Subjective health and health-related life satisfaction vary by gender. Male seniors reported better subjective health and higher life satisfaction compared to women. Additionally, subjective health improved when physical activity levels met or exceeded the recommended guidelines for adults and older individuals. These findings align with prior research indicating that the duration and intensity of exercise are important determinants of subjective health in individuals with disabilities [16,45].

Multiple regression analysis revealed that gender and disability-related factors significantly influenced health-related life satisfaction within certain age groups. Specifically, being female was a significant determinant in children/adolescents and seniors, whereas the presence of a severe disability or multiple disabilities was a significant factor in adults.

Health-related factors were consistently identified as determinants of life satisfaction across all age groups. Self-esteem influenced the health-related life satisfaction of individuals with disabilities, regardless of age. This supports the findings of a previous study involving adults with cerebral palsy who participated in sports, which indicated that higher self-esteem in disabled individuals positively impacts life satisfaction [46]. Similarly, depression influenced the health-related life satisfaction of people with disabilities in all age groups. This corroborates the results of a study on registered individuals with disabilities, which found that life satisfaction is influenced by both depression and the acceptance of disability [47]. The presence of chronic diseases was also a significant factor across all age groups. This observation is in line with earlier research suggesting that the quality of life [48] and life satisfaction [21] of disabled adults are greater in the absence of chronic diseases. Furthermore, subjective health was a key determinant at all ages, with higher levels of perceived health correlating with increased health-related life satisfaction. This finding is consistent with previous research on the life satisfaction of registered individuals with disabilities aged 18 and older [49].

This study has several limitations. First, because age from the panel data was categorized in 10-year intervals, the age groups in this study were fairly broad: children/adolescents (0–19 years), adults (20–59 years), and seniors (60 years and over). This age distribution, however, differs from the criteria set by the WHO physical activity guidelines [50], which categorize children/adolescents as 5 to 17 years, adults as 18 to 64 years, and seniors as 65 years and older. Future studies should reanalyze the amount of physical activity by classifying the ages of people with disabilities according to the WHO guidelines. Second, this study analyzed the subjective health and health-related life satisfaction of people with disabilities across all genders and ages. Further research should include other health and welfare factors related to subjective health and health-related life satisfaction that were not considered in this study.

This study is meaningful because it compared determinants of health-related life satisfaction involving sociodemographic, disability-related, and health-related factors among a broad spectrum of people with disabilities in an analysis that was stratified by gender and age groups. The observation that females with disabilities report poorer health could be attributed to several factors: women generally hold a lower socioeconomic status than men within Korean society, they may face gender discrimination rooted in Confucian cultural values, and they often carry a heavier burden of domestic responsibilities [51]. Women with disabilities have indicated that economic dependence and societal prejudice, including disregard and discrimination, are among their most significant challenges [6]. They also encounter obstacles in accessing local physical activity facilities due to a lack of assistance during exercise, insufficient information, and time constraints [52].

The results of this study also highlight a gender disparity in physical activity levels, perceived health status, and health-related life satisfaction among individuals with disabilities. Furthermore, since health-related factors may act as common determinants of health-related life satisfaction across all age groups, it is crucial to pay closer attention to these factors when devising health promotion strategies and educational programs for the disabled population. Consequently, we recommend further research into additional health-related factors that could influence the health-related life satisfaction of people with disabilities, as well as the creation of tailored health education that addresses the specific needs of different genders and age groups.

## Figures and Tables

**Table 1.** Disability-related and sociodemographic characteristics by age and gender (N=2,796)

Disability characteristics	Categories	n (%) or mean±SD									
		Total	Children/adolescents (0–19 years)			Adults (20–59 years)			Seniors (≥60 years)		
			(n=404)			(n=1,190)			(n=1,202)		
			Male	Female	χ²/t (*p*)	Male	Female	χ²/t (*p*)	Male	Female	χ²/t (*p*)
Disability type	External physical functional disability	1,807 (64.6)	178 (67.4)	89 (63.6)	0.60 (.436)	356 (54.1)	312 (58.6)	2.46 (.116)	501 (71.7)	371 (73.8)	0.63 (.425)
	Internal organic functional or mental disability	989 (35.4)	86 (32.6)	51 (36.4)		302 (45.9)	220 (41.4)		198 (28.3)	132 (26.2)	
Degree of disability	Severe	1,281 (45.8)	144 (54.5)	92 (65.7)	4.69 (.030)	349 (53.0)	268 (50.4)	0.83 (.361)	252 (36.1)	176 (35.0)	0.14 (.705)
	Mild	1,515 (54.2)	120 (45.5)	48 (34.3)		309 (47.0)	264 (49.6)		447 (63.9)	327 (65.0)	
Multiple disabilities	Yes	202 (7.2)	51 (19.3)	29 (20.7)	0.11 (.738)	45 (6.8)	20 (3.8)	5.40 (.020)	35 (5.0)	22 (4.4)	0.26 (.601)
	No	2,594 (92.8)	213 (80.7)	111 (79.3)		613 (93.2)	512 (96.2)		664 (95.0)	481 (95.6)	
Living alone	Yes	537 (19.2)	1 (0.4)	0 (0)	0.53 (.466)	166 (25.2)	88 (16.5)		152 (21.7)	130 (25.8)	
								13.22 (<.001)			2.73 (.098)
	No	2,259 (80.8)	264 (99.6)	140 (100)		492 (74.8)	444 (83.5)		547 (78.3)	373 (74.2)	
Monthly family income (1 million Korean won^†^)		2.78±2.18	4.24±2.12	3.76±1.73	2.30 (.022)	2.81±2.41	2.81±211	0.01 (.989)	239±1.94	2.21±1.97	1.63 (.103)

† One million Korean won is approximately 750 US dollars.

**Table 2.** Health-related factors and health-related life satisfaction by age and gender (N=2,796)

Variable	Categories	n (%) or mean±SD									
			Children/adolescents (n=404)			Adults (n=1,190)			Seniors (n=1,202)		
			Male	Female	χ²/t (*p*)	Male	Female	χ²/t (*p*)	Male	Female	χ²/t (*p*)
Health-related life satisfaction		5.00±2.15	6.20±2.34	6.76±2.15	–2.31 (.021)	4.93±2.20	4.89±2.06	0.35 (.721)	4.79±2.01	4.36±1.77	3.90 (<.001)
											
Amount of physical activity	Meeting or exceeding recommendations	1,472 (52.6)	32 (12.1)	13 (9.3)	0.74 (.389)	389 (59.1)	284 (53.4)	3.93 (.047)	471 (67.4)	283 (56.3)	15.46 (<.001)
	Less than	1,324 (47.4)	232 (87.9)	127 (90.7)		296 (40.9)	248 (46.6)		228 (32.6)	220 (43.7)	
	recommended										
Self-esteem		27.29±4.01	28.67±3.93	28.57±4.07	0.22 (.826)	27.08±4.25	27.04±4.01	0.18 (.852)	27.13±3.97	26.95±3.53	0.81 (.415)
Depression	Not depressed	1,589 (56.8)	194 (73.5)	101 (72.1)	0.08 (.772)	380 (57.8)	275 (51.7)	4.36 (.037)	374 (53.5)	265 (52.7)	0.07 (.778)
	Depressed	1,207 (43.2)	70 (26.5)	39 (27.9)		278 (42.2)	257 (48.3)		325 (46.5)	238 (47.3)	
Having a chronic disease	Yes	1,598 (57.2)	37 (14.0)	17 (12.1)	0.27 (.599)	341 (51.8)	317 (59.6)	7.17 (.007)	495 (70.8)	391 (77.7)	7.22 (.007)
	No	1,198 (42.8)	227 (86.0)	123 (87.9)		317 (48.2)	215 (40.4)		204 (29.2)	112 (22.3)	
Subjective health		2.60±0.57	2.98±0.48	2.99±0.44	–0.24 (.809)	2.62±0.56	2.58±0.55	1.17±.240	2.51±0.54	2.42±0.56	2.98 (.003)

**Table 3.** Differences in subjective health and health-related satisfaction across age groups by amount of physical activity (PA) and gender (N=2,796)

Variable	Amount of PA	Children/adolescents (n=404)				Adults (n=1,190)				Seniors (n=1,202)			
		Mean±SD		F (*p*)		Mean±SD		F (*p*)		Mean±SD		F (*p*)	
		Male	Female	Total PA	Sex	Male	Female	Total PA	Sex	Male	Female	Total PA	Sex
Health-related life satisfaction	Meeting or exceeding recommendations	6.75±2.38	7.08±1.97	1.94 (.164)	5.37 (.021)	5.04±2.22	5.04±2.14	5.58 (.018)	.12 (.721)	4.88±2.04	4.46±1.76	6.10 (.014)	15.246 (<.001)^†^
	Less than recommended	6.13±2.33	6.72±2.17			4.77±2.17	4.71±1.96			4.62±1.93	4.25±1.77		
Subjective health	Meeting or exceeding recommendations	3.06±0.35	3.08±0.27	1.54 (.215)	.05 (.809)	2.65±0.56	2.63±0.52	9.94 (.002)^†^	1.38 (.240)	2.52±0.54	2.47±0.55	5.25 (.022)	8.89 (.003)
	Less than recommended	2.97±0.49	2.98±0.45			2.57±0.57	2.51±0.57			2.50±0.54	2.34±0.57		

† Welch t-test.

**Table 4.** Factors affecting health-related life satisfaction by age (N=2,796)

Independent variable	Children/adolescents (n=404)						Adults (n=1,190)						Seniors (n=1,202)					
	Model 1			Model 2			Model 1			Model 2			Model 1			Model 2		
	β	t	*p*	β	t	*p*	β	t	*p*	β	t	*p*	β	t	*p*	β	t	*p*
Gender^†^	.12	2.39	.017	.10	2.56	.011	–.02	–0.93	.349	.01	0.71	.473	–.10	–3.84	<.001	–.06	–2.57	.010
Degree of disability^†^	.04	0.81	.418	.01	0.01	.988	.11	3.54	<.001	.05	2.04	.041	.14	4.79	<.001	.04	1.94	.052
Disability type^†^	.05	0.92	.358	.04	0.82	.412	.03	1.00	.314	.02	0.83	.405	–.02	–0.99	.320	–.04	–1.64	.100
Multiple disabilities^†^	.03	0.69	.486	–.02	–0.46	.644	.11	3.87	<.001	.05	2.15	.032	.09	3.18	.002	.03	1.31	.189
Living alone^†^	–.03	–0.64	.521	–.03	–0.76	.446	.07	2.33	.020	.04	1.89	.059	–.01	–0.51	.610	–.02	–0.90	.365
Mean of monthly family income	.01	0.18	.850	–.02	–0.46	.640	.12	4.06	<.001	.01	0.34	.731	.13	4.38	<.001	0.03	1.42	0.156
Self-esteem				.10	2.27	.024				.08	3.16	.002				.16	6.45	<.001
Depression^†^				–.15	–3.56	<.001				–.13	–4.98	<.001				–.11	–4.72	<.001
Having a chronic disease^†^				.12	2.82	.005				.19	7.49	<.001				.13	5.57	<.001
Amount of physical activity^†^				.03	0.75	.451				.01	0.74	.454				.01	0.36	.717
Subjective health				.42	9.69	<.001				.36	13.58	<.001				.39	15.55	<.001
F (*p*)	1.29 (.257)			16.82 (<.001)			11.15 (<.001)			55.41 (<.001)			13.37 (<.001)			58.73 (<.001)		
Adjusted R^2^	.01			.30			.04			.33			.05			.34		

† The reference values were gender (male), degree of disability (severe), disability type (external physical functional disability), multiple disabilities (yes), living alone (yes), depression (not depressed), having a chronic disease (yes), and amount of physical activity (less than recommended).
